# Weill Cornell Medicine: A History of Cornell’s Medical School

**DOI:** 10.5195/jmla.2017.218

**Published:** 2017-04

**Authors:** Danielle Becker

Having previously lived and worked on the Upper East Side of New York City for several years, I have always been interested in learning more about the history of the area. I shared a subway stop with Memorial Sloan Kettering and walked past Cornell Medical School on my way home; it was in my neighborhood. So when I had the opportunity to read and review the book *Weill Cornell Medicine: A History of Cornell’s Medical School,* I was gung-ho.

It’s not for nothing that I am a big fan of the Cinemax series, *The Knick,* which is about a fictionalized version of the Knickerbocker Hospital in New York during the early twentieth century (starring Clive Owen, directed by Steven Soderbergh, not bad credentials). I love the history of the city, and I am a hospital librarian, so discovering the history of not only Cornell’s Medical School, but also its hospital and the evolution of these institutions and their place in medical history promised to be an engaging read.

The book opens with an introduction that boldly states, “The history of Cornell University Medical College—now Weill Cornell Medicine—is essentially the history of modern medicine” (p. i). It glides through the following pages, illustrating the many ways that this statement is absolutely true. Cornell University Medical College was established in 1898, a time in which only 5% of American physicians were women (p. x), and yet, Cornell, from the first, admitted women into its program when other schools refused to. The first class of 278 students had 26 females. A year later, Elizabeth Blackwell, the first woman to receive a medical degree in the United States, founded the Women’s Medical Center of New York Infirmary for Women and Children, which then merged with Cornell University Medical Center (CUMC), resulting in adding 70 women to the student body (p. x). The first graduating class had 12 women out of 67 degree recipients.

The first chapter familiarizes the reader with the origins of medical schools in general. In 1876, there were only 76 medical schools in the United States, with many opening at a fast pace. But of those new schools, very few were connected to a university or a teaching hospital. In fact, many of these schools were owned by professors whose entrance requirements were typically lower than those of high schools. Students gained a superficial knowledge of medicine, and the only scientific course that they took was anatomy, but nonetheless, they were awarded degrees from these schools after two four-month terms, regardless of their academic performance (p. 2).

In the late 1870s, schools such as Johns Hopkins University School of Medicine, Harvard University, the University of Michigan, and the University of Pennsylvania started introducing educational reforms resulting in a dramatic shift in how the proprietary schools were operating. In 1898, Cornell proposed a new medical school that would “maintain both high academic standards and financial stability” (p. 2). Since the city was in the throes of industrialization, this proposal could not have been better timed. With industrialization came new immigrants stuffed into cramped tenement housing, since known for poor ventilation and antiquated (and in some cases nonexistent) plumbing and sewage systems. From these tenements arose infectious diseases and high mortality rates amongst residents (p. 5). The formal opening of CUMC was on October 4, 1898, with temporary housing at Loomis Laboratory on East Twenty-Sixth Street and nearby rented buildings at Bellevue Hospital. CUMC’s permanent home on First Avenue officially opened on December 29, 1900 (p. 12).

The book follows CUMC’s progression. As the country changed, CUMC changed along with it, with each chapter guiding the way through its rich history. The reader learns that CUMC graduated the school’s first African American medical student in 1915, became one of the first medical schools to provide health care for its students in 1916, and hired George Papanicolaou, whose research eventually led to the development of the “Pap smear” for cervical cancer screening in the 1920s. Readers learn about how CUMC was evolving through examples such as: “The poor, who had previously been treated at dispensaries, started to receive free care from hospital outpatient clinics. Patients who could afford better care—like the wealthy elites portrayed in F. Scott Fitzgerald’s classic novel of the decade, *The Great Gatsby*—chose private specialists” (p. 30).

But the working and middle classes were unable to afford health care, so in 1921 Cornell stepped up and created the Cornell Pay Clinic to offer specialized care to these populations at reasonable prices (p. 30). Chapters two and three document partnerships and the move to the Upper East Side. Chapters four and five discuss the CUMC during World War II and the postwar boom. By 1935, the country was spending 4% of its gross national product (GNP) on health care, which was far higher than it was at the turn of the century (p. 79). From this situation sprung the New Deal, which began debates on health care coverage, that were not to be solved until the passage of the Social Security Amendments of 1965 and the creation of the Medicare and Medicaid programs (p. 124).

The work marches forward chronologically through the social activism of the 1960s, when medical schools and teaching hospitals were addressing the needs of their surrounding communities by actively engaging in outreach efforts to underserved patients (p. 121). The response to health care changes that brought about the Cornell Medical Group in 1967, which afforded Medicare and Medicaid patients the status and benefits of semiprivate patients so that they, too, could be treated by physician teams and given access to the same facilities and care as semiprivate patients (p. 125).

The last three chapters explore the next three decades and into the twenty-first century. A hundred years after its founding, Cornell University Medical College was renamed Weill Cornell Medical College in 1998. Since then, the school has been growing by not only improving and adding new space to its facilities, but also by forming the New York Hospital Care Network, which links affiliating hospitals, clinics, and nursing homes throughout the New York Metro area (p. 201).

The authors do a fair and objective job of detailing the history of the CUMC, without including only aggrandizing details. Instead, they report the historical facts and characters involved without bias (sometimes seen in books of this nature). I learned about how hospitals and medical schools work in unison and how they work to make it through the often turbulent and unforgiving times that we live in. I found it interesting that partnerships had been forged and met with successes and failures, but that never stopped CUMC from moving forward. Another appropriate feature of this work is the inclusion of notes at the back of the book that are easily accessible to a common reader. There is also a bibliography for future reading and a handy index for quick reference. One thing I did not find very interesting were the cutouts in the chapters detailing the characters such as administrators, founders, and faculty that were referenced throughout. Although helpful to the researcher, as a lay reader, I found them to be rather dull in both content and tone. Unfortunately, I think the layout and the book’s design are greatly lacking and suffer from creative drought. For example, I can only imagine the wonderful photographs and art that exist in the historical archives of Cornell’s Medical School. Why not include these resources?

I believe Laurie H. Glimcher captured the book’s value best in her introduction: “Weill Cornell Medicine is a place where the medicine of tomorrow is happening today. As we plan for the future, it is well to remember the lessons of the past. You will find them here, in this wonderful book” (p. xvii). It is an appealing read for anyone who is interested in the origins of medical education in this country or for someone who is exploring how teaching hospitals and medical schools relate to the history of New York City (serving as an example for any large urban area) and the country overall. *Weill Cornell Medicine: A History of Cornell’s Medical School* is a thorough book whose core audience is likely academic at the undergraduate and graduate levels.

